# Protease-activated receptor 2 (PAR-2) antagonist AZ3451 as a novel therapeutic agent for osteoarthritis

**DOI:** 10.18632/aging.102586

**Published:** 2019-12-16

**Authors:** Xiaojian Huang, Bowei Ni, Yang Xi, Xiangyu Chu, Rui Zhang, Hongbo You

**Affiliations:** 1 Department of Orthopedics, Tongji Hospital, Tongji Medical College, Huazhong University of Science and Technology, Wuhan, Hubei 430030, China

**Keywords:** osteoarthritis, PAR2, AZ3451, autophagy, apoptosis

## Abstract

Osteoarthritis (OA) is a highly prevalent joint disorder blamed for pain and disability in older individuals. It’s commonly accepted that inflammation, apoptosis, autophagy and cellular senescence participate in the progress of OA. Protease activated receptor 2 (PAR2), a member of the G-protein coupled receptors, is involved in the regulation of various inflammation diseases. Previous studies have identified PAR2 as a potential therapeutic target for the treatment of OA. Here, we investigated the role of PAR2 antagonist AZ3451 in inflammation response, apoptosis, autophagy and cellular senescence during OA. We confirmed that PAR2 expression was significantly up-regulated in OA articular cartilage tissues as well as in interleukin 1β (IL-1β) stimulated chondrocytes. We demonstrated AZ3451 could prevent the IL-1β-induced inflammation response, cartilage degradation and premature senescence in chondrocytes. Further study showed that AZ3451 attenuated chondrocytes apoptosis by activating autophagy in vitro. The P38/MAPK, NF-κB and PI3K/AKT/mTOR pathways were involved in the protective effect of AZ3451. In vivo, we found that intra-articular injection of AZ3451 could ameliorate the surgery induced cartilage degradation in rat OA model. Our work provided a better understanding of the mechanism of PAR2 in OA, and indicated that PAR2 antagonist AZ3451 might serve as a promising strategy for OA treatment.

## INTRODUCTION

Osteoarthritis (OA) is the most prevalent form of arthritis and has attracted widespread interest among clinicians in recent decades [1]. It is a leading cause of chronic pain and impaired mobility in older individuals [2]. As an age related disease, OA affects 240 million people globally, approximately 10% of men and 18% of women over 60 years old, and significantly affects the quality of life and healthcare in older population [3]. Currently, most of therapeutic strategies designed for OA are focused on relieving inflammation and pain [4]. In addition to joint replacement surgery, osteoarthritis is commonly considered as an incurable disease. Therefore, exploring the pathogenesis of osteoarthritis is critical for OA treatment.

Loss of cartilage integrity and chondrocytes senescence are the features of OA [5]. Excessive release of inflammatory factors including interleukin 1β (IL-1β), tumor necrosis factor (TNF) α, cyclooxygenase-2 (COX-2) and inducible nitric oxide synthase (iNOS), induces the expression of proteolytic enzymes such as matrix metalloproteinases (MMPs) and a disintegrin and metalloproteinase with thrombospondin motifs (ADAMTS), thus leading to the loss of cartilage [6]. Cellular senescence is a state of irreversible cell cycle arrest. Senescent chondrocytes lose the ability to maintain and repair tissue, thus increasing the risk of cartilage degeneration [7]. Another major characteristic of osteoarthritis is cell decrease, which is mainly caused by programmed cell death- apoptosis [8]. Chondrocytes, the sole cellular constituents of normal cartilage in mammals, are essential for the maintenance of the cartilage homeostasis [9]. Thus, the survival of the chondrocytes is crucial for maintaining a suitable cartilage matrix. Apoptosis has been shown to be related to the severity of matrix depletion and cartilage destruction in osteoarthritic tissue [10]. Autophagy, also known as type II programmed cell death, has gained increasing attention in OA [11]. It is a highly conserved homeostatic process that degrades cytosolic macromolecules and organelles to maintain cellular homeostasis and quality control [12]. It is widely accepted that autophagy is a constitutively active and apparently protective process for maintaining cartilage homeostasis [13].

Protease-activated receptor 2 (PAR-2) is a member of the seven-transmembrane G protein-coupled receptor family (30700181). It is involved in the pathogenesis of various diseases including inflammatory, gastrointestinal, respiratory and metabolic diseases [14]. In vitro, PAR2 agonist increased inflammation in human kidney tubular epithelial cells [15]. Activation of the PAR2 might lead to the secretion of inflammatory cytokines IL-6, IL-8 and IL-1β in peripheral blood monocytes [16]. Functional inhibition of PAR2 alleviated allergen-induced airway hyper-responsiveness and inflammation in mice [17]. Previous studies have identified that PAR2-deficient mice (PAR2−/−) were significantly protected from cartilage damage in experimental OA generated by destabilization of the medial meniscus (DMM) [18]. The level of PAR2 in OA chondrocytes was much higher than in normal chondrocytes [19]. However, the detailed mechanism of PAR2 in OA remains unclear. In the present study, we proposed PAR2 antagonist AZ3451 as a promising therapy for OA and explored the underlined mechanism.

## RESULTS

### PAR2 is highly expressed in rat OA cartilage tissue and in IL-1β treated chondrocytes

**Figure 1:**
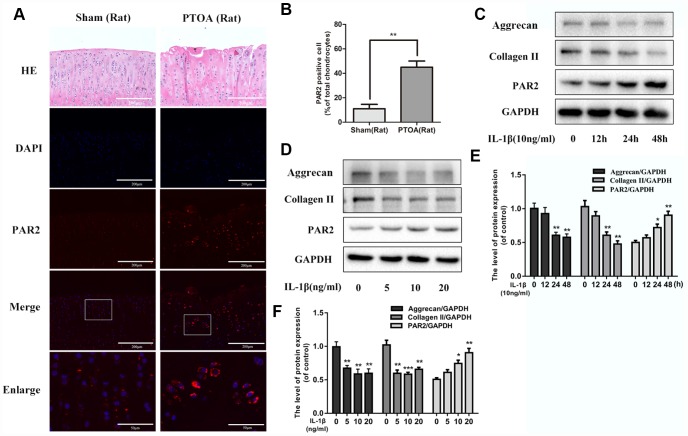
**PAR2 is highly expressed in rat OA cartilage tissue and in IL-1β treated chondrocytes.** (**A**) Representative H&E staining and immunofluorescence staining of PAR2 in rat knee articular cartilage from sham and PTOA model. (**B**) Quantitation of immunofluorescence staining of PAR2. (**C**, **D**) Representative western blots and quantification data of PAR2, Collagen II and Aggrecan in chondrocytes after stimulation with various dose of IL-1β for 24h. (**E**, **F**) Representative western blots and quantification data of PAR2, Collagen II and Aggrecan in chondrocytes after stimulation with 10ng/ml IL-1β under different time course. Data are shown as the mean ± SD. Significant differences between groups are indicated as ***P <0.001, **P < 0.01 and *P < 0.05.

To investigate the change of PAR2 level in OA development, we detected the difference in PAR2 expression between normal and PTOA rat cartilage by immunofluorescence staining. We observed the percentage of PAR2 positive chondrocytes was significantly increased in rat OA cartilage, in comparison to normal cartilage (Figure 1A, 1B). In addition, we exposed chondrocytes with IL-1β to mimic OA model in vitro and detect the PAR2 and collagen II protein levels change. As shown in Figure 1C–1F, IL-1β lead to the degradation of collagen II as well as aggrecan, and increased PAR2 expression in a time- and dose-dependent manner in rat chondrocytes.

### PAR2 antagonist AZ3451 suppresses IL-1β-induced inflammation response, cartilage degradation and premature senescence

**Figure 2:**
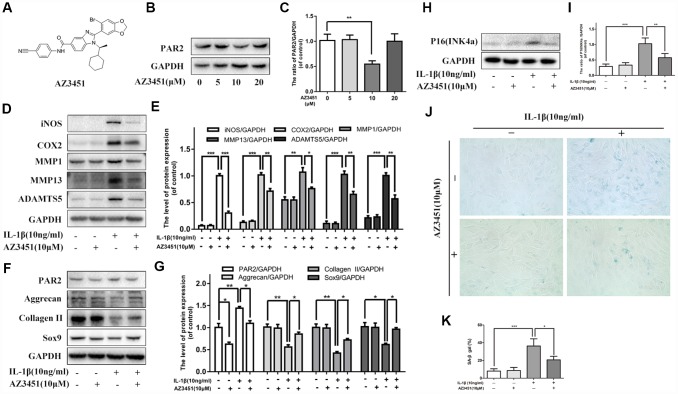
**PAR2 antagonist AZ3451 suppresses IL-1β-induced inflammation response, cartilage degradation and premature senescence in chondrocytes.** (**A**) Chemical structure of AZ3451. (**B**, **C**) Representative western blots and quantification data of PAR2 in chondrocytes of each group. (**D**, **E**) Representative western blots and quantification data of iNOS, COX2, MMP1, MMP13 and ADAMTS5 in chondrocytes of each group. (**F**, **G**) Representative western blots and quantification data of PAR2, Aggrecan, Collagen II and Sox9 in chondrocytes of each group. (**H**, **I**) Representative western blots and quantification data of p16INK4a in chondrocytes of each group. (**J**, **K**) SA-β gal staining assay was performed in chondrocytes as treated above. Data are shown as the mean ± SD. Significant differences between groups are indicated as ***P <0.001, **P < 0.01 and *P < 0.05.

Given that PAR2 expression was up-regulated in OA articular cartilages, we further explored whether PAR2 antagonist could cure osteoarthritis in vitro. AZ3451, as shown in Figure 2A, is a potent antagonist which can bind to a remote allosteric site outside the helical bundle of the PAR2 [20]. We first detect the inhibitory effect of AZ3451 on PAR2 expression in chondrocytes. As shown in Figure 2B, 2C, 10 μM AZ3451 can effectively inhibit PAR2 expression in chondrocytes. To investigate the mechanism underlying the effects of PAR2 antagonist in OA, chondrocytes were stimulated with AZ3451 (10 μM) and IL-1β (10 ng/ml) for 48 h. As shown in Figure 2D, 2E, IL-1β increased the inflammatory cytokines and catabolic genes expression, including iNOS, COX2, MMP1, MMP13 and ADAMTS5, while treatment with AZ3451 alleviated this process. Furthermore, decreased Collagen II, aggrecan and SOX9 expressions induced by IL-1β were also inhibited by AZ3451 (Figure 2F, 2G). The cellular senescence level was detected by SA-β-gal staining and p16INK4a protein level. As shown in Figure 2H–2K, the IL-1β-treated chondrocytes exerted higher SA-β-gal activity and p16INK4a protein expression compared with the control group, while PAR2 antagonist AZ3451 significantly prevented this process.

### AZ3451 alleviates the IL-1β-induced autophagy downregulation in chondrocytes

**Figure 3:**
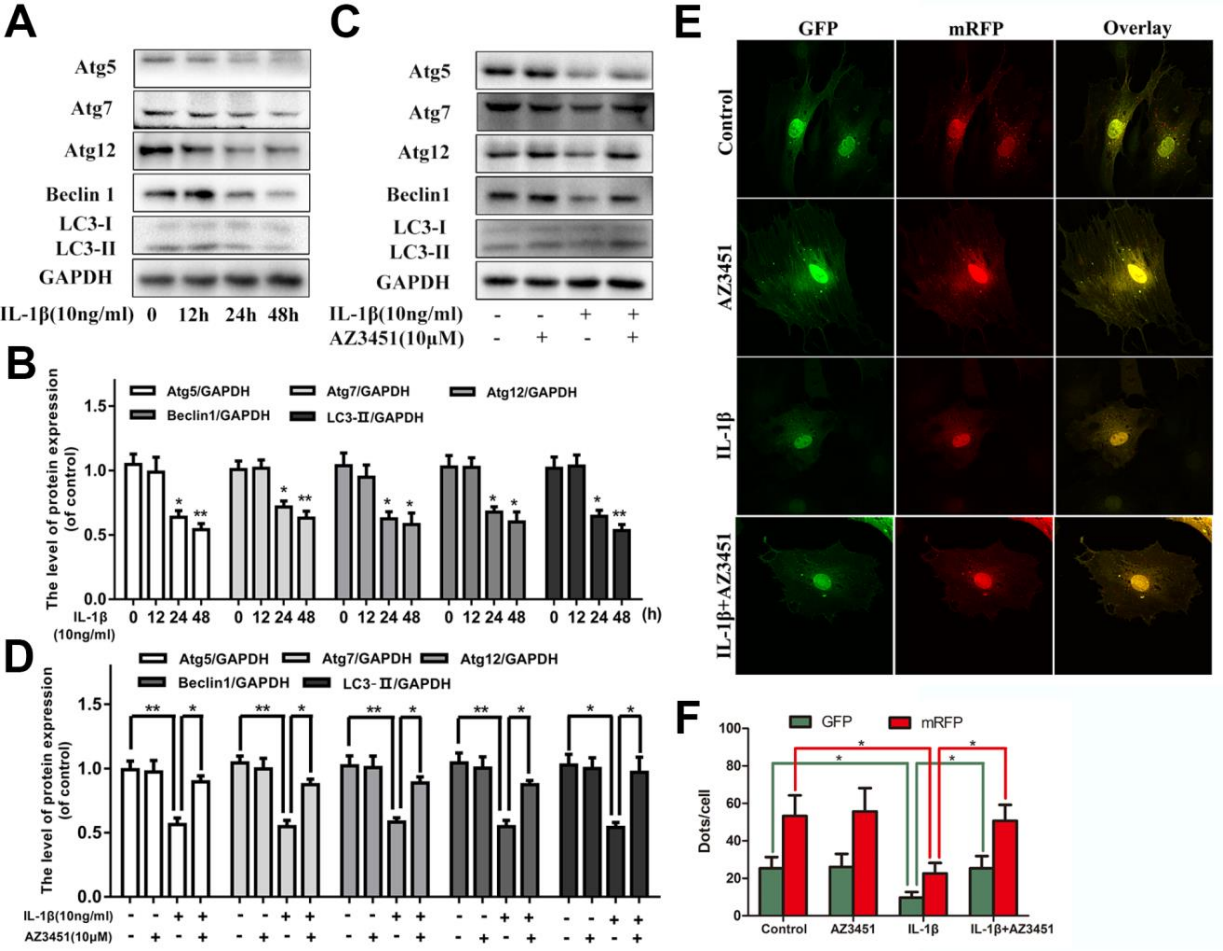
**AZ3451 alleviates the IL-1β-induced autophagy downregulation in chondrocytes.** (**A**, **B**) Representative western blots and quantification data of Atg5, Atg7, Atg12, Beclin1 and LC3 in chondrocytes after stimulation with 10ng/ml IL-1β under different time course. (**C**, **D**) Representative western blots and quantification data of Atg5, Atg7, Atg12, Beclin1 and LC3 in chondrocytes as treated above. (**E**, **F**) Fluorescence microscopy analysis of chondrocytes transfected with mRFP-GFP-LC3 adenovirus in each group. Data are shown as the mean ± SD. Significant differences between groups are indicated as ***P <0.001, **P < 0.01 and *P < 0.05.

Increasing evidences indicated that autophagy was an important protective mechanism in OA development. We next verified whether PAR2 antagonist AZ3451 could regulate autophagy processes in chondrocytes. As shown in Figure 3A, 3B, IL-1β decreased the autophagy markers (Atg5, Atg7, Atg12, Beclin1 and LC3) expression in a time-dependent manner. While AZ3451 treatment remarkably reversed the decreased autophagy related genes expression induced by IL-1β (Figure 3C, 3D). In addition, the tandem GFP-RFP-LC3 adenovirus was utilized to confirm autophagy induction by form punctate that represented autophagosome formation. As shown in Figure 3E, 3F, after infection with the GFP-RFP-LC3 adenovirus, there was fewer red puncta under IL-1β stimulation in chondrocytes; However, AZ3451 treatment cells presented more red puncta. These results demonstrated that AZ3451 could enhance autophagosome conversion to autophagolysosomes, and thus promoting autophagy flux and autophagy level.

### AZ3451 attenuates IL-1β induced apoptosis in chondrocytes

**Figure 4:**
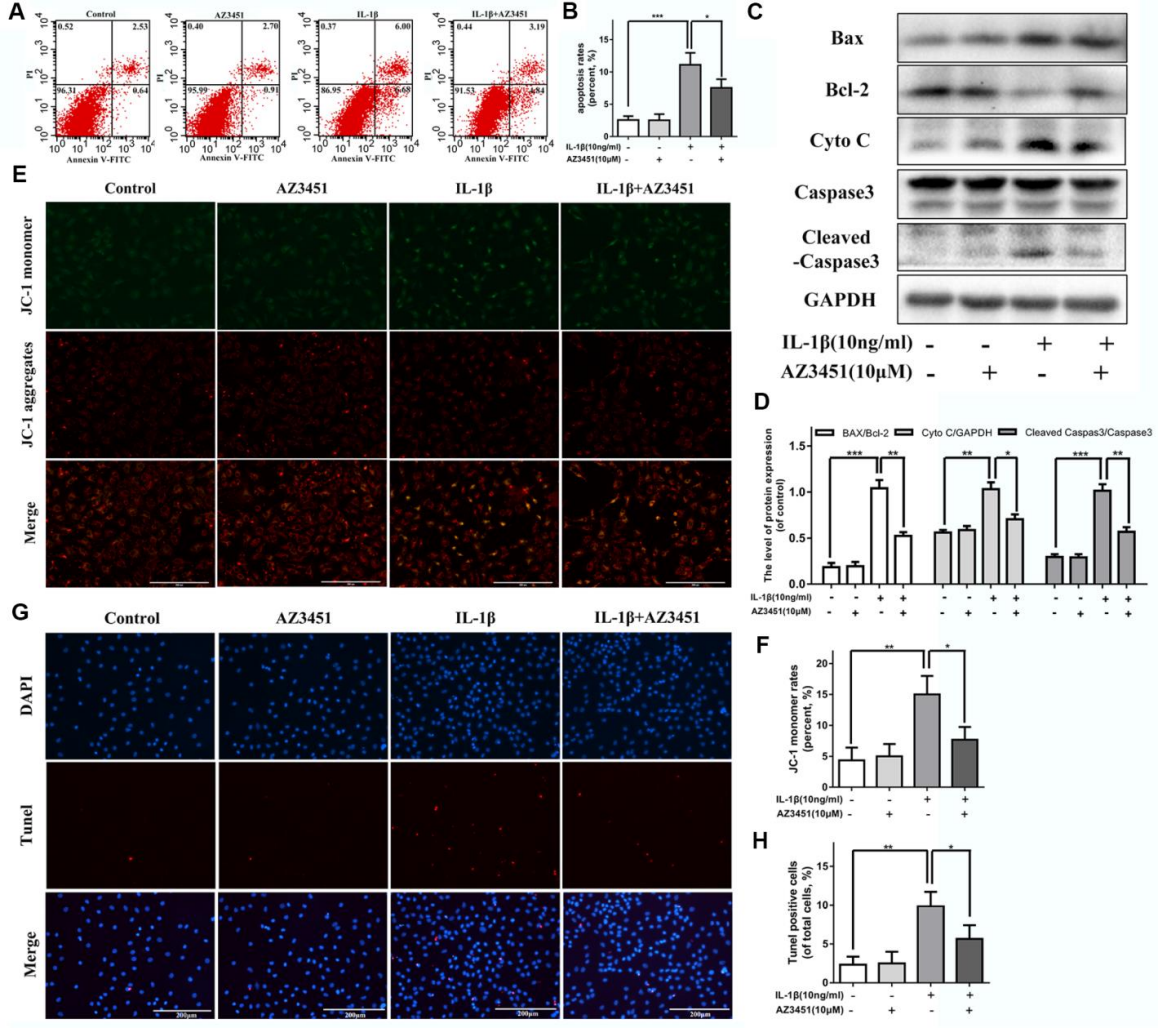
**AZ3451 attenuates IL-1β induced apoptosis in chondrocytes.** (**A**, **B**) Flow cytometry of Annexin V-FITC/PI staining and quantification data of the apoptosis rates in chondrocytes as treated above. (**C**, **D**) Representative western blots and quantification data of Bcl-2, BAX, Cyto C and Cleaved caspase 3 in chondrocytes as treated above. (**E**, **F**) Representative fluorescence photographs of mitochondrial membrane potential and JC-1 monomer rates in chondrocytes as treated above. (**G**, **H**) TUNEL staining assay was performed in chondrocytes as treated above. Data are shown as the mean ± SD. Significant differences between groups are indicated as ***P <0.001, **P < 0.01 and *P < 0.05.

To detect the role of PAR2 antagonist AZ3451 on IL-1β induced apoptosis, the Annexin V-FITC/PI staining and flow cytometric analysis were used to measure apoptosis rates in chondrocytes. As shown in Figure 4A, 4B, AZ3451 treatment caused a marked decrease of apoptotic chondrocytes induced by IL-1β. The apoptosis related genes expression such as Bcl-2, BAX, Cyto C and Cleaved caspase 3 were detected by western blot. We found AZ3451 downregulated the IL-1β-induced increasing Cyto C, Cleaved caspase 3 expression and reversed the BAX/Bcl-2 ratio in chondrocytes (Figure 4C-D). Decreased mitochondrial membrane potential is one of the characteristics of apoptotic cells. As shown in Figure 4E, 4F, IL-1β stimulation decreased the mitochondrial membrane potential, while AZ3451 treatment remarkably reversed this process. A TUNEL assay was performed to detected DNA damage. We found that AZ3451 treatment downregulated the IL-1β-induced increasing levels of TUNEL positive cells (Figure 4G, 4H).

### CQ attenuates the anti-apoptotic effect of AZ3451 in chondrocytes

**Figure 5:**
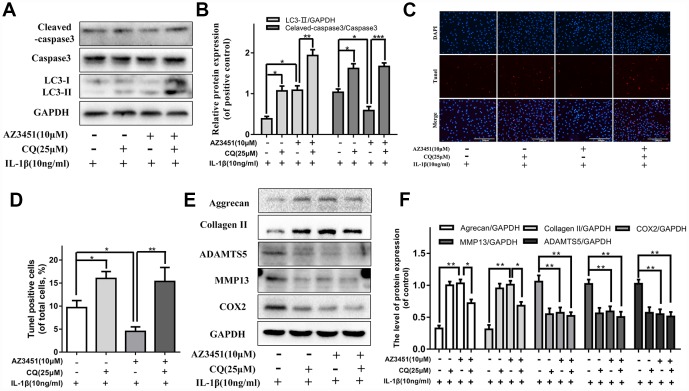
**CQ attenuates the anti-apoptotic effect of AZ3451 in chondrocytes.** (**A**, **B**) Representative western blots and quantification data of Cleaved caspase 3 and LC3 in chondrocytes as treated above. (**C**, **D**) TUNEL staining assay was performed in chondrocytes as treated above. (**E**, **F**) Representative western blots and quantification data of collagen II, aggrecan, COX2, ADAMTS5 and MMP13 as treated above. Data are shown as the mean ± SD. Significant differences between groups are indicated as ***P <0.001, **P < 0.01 and *P < 0.05.

To investigate how autophagy interacted with anti-apoptotic effects of AZ3451, chloroquine (CQ), an autophagylysosome pathway inhibitor, was used to inhibit the downstream of autophagy flux in chondrocytes. As shown in Figure 5A, 5B, western blotting results showed that the IL-1β-mediated upregulation of cleaved caspase 3 was significantly attenuated by AZ3451, while inhibition of autophagy with CQ reversed the anti-apoptotic effect of AZ3451. Similarly, the reduction of the percentage of TUNEL positive cells was also markedly attenuated by CQ (Figure 5C, 5D). Taken together, the results indicated that autophagy was essential for the anti-apoptotic effect of AZ3451. To explore whether autophagy was associated with AZ3451-mediated chondrocyte metabolism, we measured the levels of main degeneration associated proteins (Aggrecan, Collagen, ADAMTS5, MMP13 and COX2). As shown in Figure 5E, 5F, CQ treatment decreased the increased the level of COX2, MMP13, ADAMTS5 and attenuated the degradation of collagen II and aggrecan induced by IL-1β. Combined AZ3451 and CQ treatment couldn’t inhibit the protective effect of AZ3451 in IL-1β induced catabolic factors (COX2, MMP13 and ADAMTS5). While we observed that the AZ3451 decreased degradation of collagen II and aggrecan was partially weakened in the presence of CQ. The results indicated that inhibition of autophagy with CQ weakened AZ3451-mediated prevention of cartilage matrix degradation without affected AZ3451-mediated catabolic processes.

### AZ3451 attenuates the activation of the P38/MAPK, NF-κB and PI3K/AKT/mTOR pathways induced by IL-1β in chondrocytes

**Figure 6:**
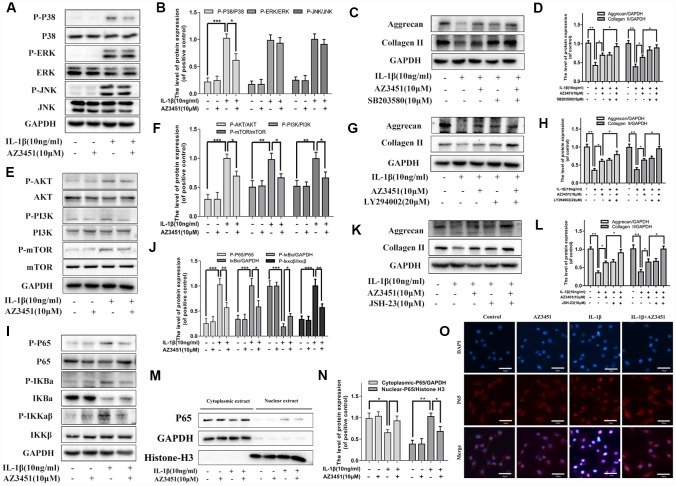
**AZ3451 attenuates the activation of the P38/MAPK, NF-κB and PI3K/AKT/mTOR pathways induced by IL-1β in chondrocytes.** (**A**, **B**) Representative western blots and quantification data of MAPK pathways in chondrocytes as treated above. (**C**, **D**) Representative western blots and quantification data of collagen II and aggrecan as treated above. (**E**, **F**) Representative western blots and quantification data of PI3K/AKT/mTOR pathways as treated above. (**G**, **H**) Representative western blots and quantification data of collagen II and aggrecan as treated above. (**I**, **J**) Representative western blots and quantification data of NF-κB pathways as treated above. (**K**, **L**) Representative western blots and quantification data of collagen II and aggrecan as treated above. (**M**, **N**) Representative western blots and quantification data of cytoplasmic P65 and nuclear P65 as treated above. (**O**) Immunofluorescence staining of the translocation of P65 in nuclear as treated above. Data are shown as the mean ± SD. Significant differences between groups are indicated as ***P <0.001, **P < 0.01 and *P < 0.05.

To further analyzing the functionary mechanism of PAR2 antagonist AZ3451 underlying OA, we detected the changes of three main signaling involved in OA, including the MAPK, NF-κB and PI3K/AKT/mTOR pathways. As shown in Figure 6, the three signaling molecules were obviously activated with IL-1β treatment. However, AZ3451 treatment suppressed IL-1β-induced P38 phosphorylation without repression of the ERK and JNK phosphorylation (Figure 6A, 6B). P38/MAPK inhibitor SB203580 treatment attenuated the degradation of collagen II and aggrecan induced by IL-1β, and combined SB203580 and AZ3451 treatment exerted better protective effects in prevention of cartilage matrix degradation compared with AZ3451 treatment alone (Figure 6C, 6D). Moreover, IL-1β-induced PI3K, AKT and mTOR phosphorylation were also inhibited by AZ3451 (Figure 6E, 6F). PI3K/AKT inhibitor LY294002 treatment attenuated the degradation of collagen II and aggrecan induced by IL-1β, and combined LY294002 and AZ3451 treatment exerted better protective effects in prevention of cartilage matrix degradation compared with AZ3451 treatment alone (Figure 6G, 6H). The phosphorylation of IKKα/β, IκBα and p65 were also significantly down-regulated by AZ3451 treatment (Figure 6I, 6J). NF-κB inhibitor JSH-23 treatment also attenuated the degradation of collagen II and aggrecan induced by IL-1β, and combined JSH-23 and AZ3451 treatment exerted better protective effects in prevention of cartilage matrix degradation compared with AZ3451 treatment alone (Figure 6K, 6L). Treatment with AZ3451 inhibited the translocation of p65 subunits into the nucleus induced by IL-1β in chondrocytes (Figure 6M–6O).

### AZ3451 rescues cartilage destruction in rat OA model in vivo

**Figure 7:**
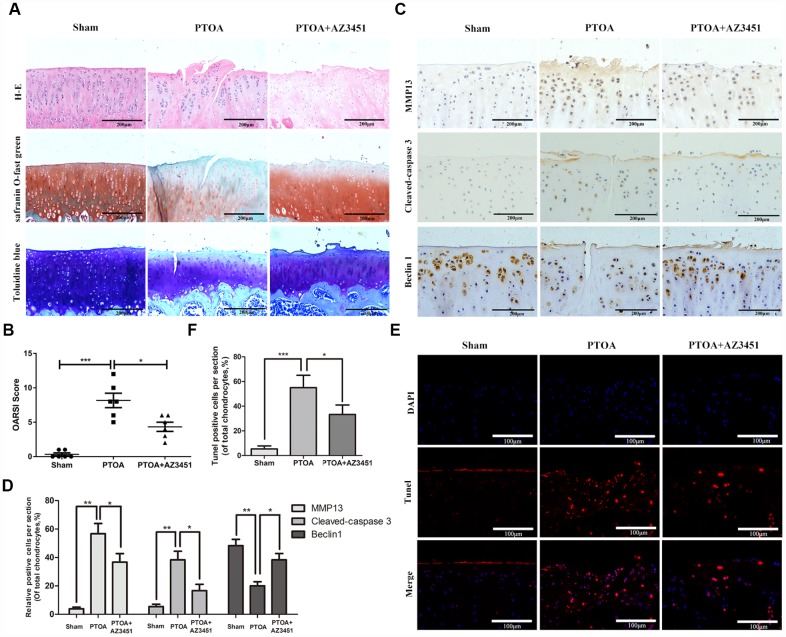
**AZ3451 rescues cartilage destruction in rat OA model in vivo.** (**A**) Representative H&E, Toluidine blue and Safranin O/Fast Green staining of cartilage in three groups after 8-week post-surgery. (**B**) OARIS scores of cartilage in three groups. (**C**, **D**) Immunohistochemical staining assay of MMP13, Cleaved-caspase3 and Beclin1 in the rat cartilage. (**E**, **F**) TUNEL staining assay in the rat cartilage. Data are shown as the mean ± SD. Significant differences between groups are indicated as ***P <0.001, **P < 0.01 and *P < 0.05.

To investigate the effect of AZ3451 on the progression of OA in vivo, AZ3451 was injected into the knee joint of the rat OA model. Safranin-O staining was performed to access histomorphology differences among rat joints. As shown in Figure 7A, we observed the significant superficial articular cartilage erosion as well as the loss of proteoglycan in the PTOA group. In contrast, AZ3451 treatment group exerted the less cartilage damage and the richer proteoglycan compared to the PTOA group. The OARSI score also showed that AZ3451 intra-articular injection significantly alleviated OA progression (Figure 7B). We then determined the expression of MMP13, Cleaved-Caspase3 and Beclin1 in each group by immunohistochemistry. Consistent with vitro results, AZ3451 could increase the Beclin1 expression and decrease MMP13 and Cleaved-Caspase 3 expression in rat OA cartilage (Figure 7C, 7D). The apoptosis of chondrocytes in cartilage was estimated by TUNEL staining. The results showed that the PTOA group exerted higher proportion of chondrocytes apoptosis, while AZ3451 treatment reversed such pathological change (Figure 7E, 7F).

## DISCUSSION

Protease activated receptor 2 (PAR2), a member of the G-protein coupled receptors, is widely distributed throughout the body and plays a crucial role in the regulation of inflammation diseases [21]. Early studies reported that PAR2 expression was up-regulated by inflammatory mediators such as TNF-α, IL-1α and lipopolysaccharide (LPS) [22]. A growing body of evidences suggested that inflammation played a crucial role in OA pathogenesis [23]. Previous studies reported that PAR2-deficient mice exerted less cartilage degradation in experimental osteoarthritis [18, 24]. Overexpression of PAR-2 in OA chondrocytes is upregulated by proinflammatory cytokines IL-1β and TNF-α [19]. In this study, we confirmed that PAR2 expression was significantly up-regulated in OA articular cartilage tissues in vivo. Moreover, we found IL-1β increased PAR2 expression in a time- and dose-dependent manner in rat chondrocytes in vitro.

Progressive degeneration of articular cartilage leads to the clinical syndrome of OA [25]. Type II collagen and aggrecan are the major structural protein of the cartilage and provide a tensile meshwork for cartilage [26]. During OA, inflammatory response proteins such as IL-1β and TNF α are over secreted by chondrocytes [26]. These proteins also stimulate the production of MMPs and ADAMTS5, enzymes that can degrade the components of cartilage [27]. Cellular senescence, which is defined as the loss of the ability of cells to divide, has also played a significant role in the pathology of OA [7]. Senescent cells contribute to tissue degeneration by sustaining chronic inflammation and extracellular matrix remodeling [28]. Aging cells can be identified using an association of multiple markers including SAβ-gal staining and the upregulated p16INK4A expression [29]. Previous studies have indicated that chondrocytes senescence contribute to the risk of cartilage degeneration by decreasing the ability of cells to maintain and repair tissue [30]. It was reported that activation of PAR2 in human osteoarthritic cartilage upregulated catabolic genes like MMPs and COX2 [31]. In the present study, we proposed PAR2 antagonist AZ3451 as a promising therapy for OA. Our data found that PAR2 antagonist AZ3451 could decrease IL-1β-induced inflammation response such as iNOS, COX2, MMP1, MMP13 and ADAMTS5 in chondrocytes. Moreover, the degradation of cartilage matrix such as collagen II and aggrecan was also suppressed by AZ3451. The IL-1β promoted generation of p16INK4A proteins and SA-β-gal stained chondrocytes were also attenuated. Taken together, these results demonstrated PAR2 antagonist AZ3451 could prevent the IL-1β-induced inflammation response, cartilage degradation and premature senescence in rat chondrocytes in vitro.

Apoptosis clearly occurs in osteoarthritic cartilage, and the compromising of chondrocyte function and survival lead to the damage of the articular cartilage [10]. It is positively correlated with the severity of cartilage destruction and matrix depletion in human osteoarthritic cartilage specimens [32]. IL-1β has been shown to induce chondrocyte apoptosis in human chondrocytes [33]. Autophagy is a protective mechanism in normal cartilage, and normal human cartilage expresses high levels of autophagy regulators including Beclin1 and LC3. In contrast, a reduction and loss of Beclin1 and LC3 expression was demonstrated in human OA cartilage and surgically induced OA cartilage in mice [34]. Pharmacological activation of autophagy by intra-articular injection of mTOR inhibitor can reduce degeneration of articular cartilage in an animal OA model [35]. Growing evidences indicate that autophagy and apoptosis have complicated intricate relationships [36]. In the present study, we found AZ3451 treatment efficiently increased the levels of autophagy-related proteins such as, Atg5, Atg7, Atg12, Beclin1 and LC3, which were decreased by IL-1β stimulation. AZ3451 also enhanced autophagosome conversion to autophagolysosomes, which ultimately restored the IL-1β-induced disruption of autophagy flux. Moreover, we found AZ3451 treatment efficiently decreased the apoptosis chondrocytes rates induced by IL-1β and suppressed the increased levels of apoptosis-related proteins, including Cyto C, Cleaved caspase 3 and reversed the increased BAX/Bcl-2 ratio. AZ3451 treatment also downregulated the IL-1β-induced increasing levels of TUNEL positive cells, and increased the mitochondrial membrane potential which were decreased by IL-1β stimulation. To further determine the relationship between autophagy and apoptosis, we used CQ (a pharmacological inhibitor of autophagy) for experiment. Our data found that AZ3451 treatment couldn’t decrease the IL-1β-induced increasing levels of TUNEL positive cells and protein level of cleaved caspase 3 in the presence of CQ, indicating that autophagy was essential for the anti-apoptotic effect of AZ3451. Taken together, these results demonstrated PAR2 antagonist AZ3451 attenuated IL-1β induced apoptosis by activating autophagy in chondrocytes. To explore whether autophagy was associated with AZ3451-mediated chondrocytes metabolism, we measured the levels of main degeneration associated proteins. Combined AZ3451 and CQ treatment couldn’t inhibit the protective effect of AZ3451 in IL-1β induced catabolic factors (COX2, MMP13 and ADAMTS5), while the decreased degradation of collagen II and aggrecan was partially weakened in the presence of CQ. The results indicated that inhibition of autophagy with CQ weakened AZ3451-mediated prevention of cartilage matrix degradation without affected AZ3451-mediated catabolic processes. This result may be related to the loss of anti-apoptotic effect of AZ3451 in the presence of CQ.

The three main signaling pathways, including the MAPK, NF-κB and PI3K/AKT/mTOR pathways, are generally thought to be responsible for the OA onset and development [37]. Inhibition of MAPK pathways abrogated proteolytic cartilage degradation by blocking MMP synthesis and activity [38]. p38/MAPK is the most critical gene which plays direct role in the induction of senescence [39]. NF-κB pathways directly or indirectly induces the expression of matrix-degrading enzymes and other OA-associated factors [40]. NF-κB inhibitors treatment were able to reduce IL-1β-induced catabolic gene expression [41]. The PI3K/AKT/mTOR pathway is a crucial intracellular signaling pathway regulating apoptosis and autophagy. mTOR is a major negative regulator of the autophagy process and a downstream target of the PI3K/AKT pathway [42]. It has also been shown that upregulation mTOR expression in OA cartilage is accompanied with an increased rate of chondrocyte apoptosis [43]. Our results showed that the activation of the P38/MAPK, NF-κB and PI3K/AKT/mTOR pathways were blocked by AZ3451 treatment, indicating that these pathways were involved in the protective effects of AZ3451 in OA.

In the animal model, we found that intra-articular injection of AZ3451 attenuated cartilage destruction notably and markedly decreased the cartilage destruction enzymes MMP13, suppressed the apoptosis marker cleaved-caspase3, and upregulated the expression of autophagy marker like Beclin1. TUNEL results in the cartilage tissues also revealed that AZ3451 inhibited apoptosis in articular chondrocytes in vivo. These in vivo data further validated the protective effect of PAR2 antagonist AZ3451 in OA.

In summary, our results confirmed that PAR2 expression was significantly up-regulated in OA. PAR2 antagonist AZ3451 could prevent the IL-1β-induced inflammation response, cartilage degradation and premature senescence in chondrocytes. Besides, PAR2 antagonist AZ3451 attenuated IL-1β induced apoptosis by activating autophagy in chondrocytes. Moreover, we found that the P38/MAPK, NF-κB and PI3K/AKT/mTOR pathways might participate in the protective effect of AZ3451. Intra-articular injection of AZ3451 notably attenuated cartilage destruction in a rat OA model. Collectively, these findings provided a better understanding of the mechanism of PAR2 in OA, and indicated that PAR2 antagonist AZ3451 may serve as a promising strategy for OA treatment.

## MATERIALS AND METHODS

### Reagents

Recombinant rat IL-1β (501-RL-010) was obtained from R&D systems (Minneapolis, MN, USA). Antagonist AZ3451 was obtained from MedChem Express (Monmouth Junction, NJ, USA). The p38 MAPK inhibitor SB203580, the NF-κB nuclear translocation inhibitor JSH-23 and the P13K/AKT inhibitor LY294002 were obtained from Selleck chemicals (Houston, TX, USA). The antibodies against P38(#8690), phospho-P38(#4511), ERK ½ (#4695), phospho-ERK ½ (#4370), JNK (#9258), phospho-JNK (#9255), phospho-P65(#3033), phospho-IKBα (#2859), phospho–IKKαβ (#2697), IKKβ (#8943), AKT (#4685), Phospho-AKT (#4060), Phospho–PI3K (#17366), mTOR (#2983), Phospho-mTOR (#5536), COX2(#12282), Atg5 (#12994), Atg7(#8558), Atg12(#4180), Beclin1(#3495), LC3A/B (#12741), Cleaved Caspase-3 (#9664) and p16INK4A (#80772) were purchased from Cell Signaling Technology (Danvers, MA). Antibody against Collagen Type II(15943-1-AP), MMP1 (10371-2-AP), IKBα (10268-1-AP), P65 (10745-1-AP), Histone-H3(17168-1-AP), Cytochrome C (10993-1-AP), Caspase 3 (19677-1-AP), Bax (50599-2-Ig), Bcl-2 (12789-1-AP) and PI3K (20584-1-AP) were purchased from Proteintech Group (Wuhan, China). Antibodies against iNOS (ab3523), MMP13 (ab39012), PAR2 (ab180953), SOX9 (ab185966) and Aggrecan (ab36861) were supplied by Abcam (Cambridge, UK). Antibodies against ADAMTS5 (BA3020) and GAPDH (BM3876) were obtained from Boster (Wuhan, China).

### Cell culture and treatments

All animal experiment processes were approved by the Experimental Animal Ethics Committee of Tongji Medical College, Huazhong University of Science and Technology (Wuhan, China). Primary rat chondrocytes were derived from the knee joint cartilage tissue of 1-week-old Sprague–Dawley rats. Cartilage pieces were sequential digested with 0.2% trypsin and 0.25% collagenase II dissolved in Dulbecco’s Modified Eagle Medium (DMEM) culture medium. The released chondrocytes were collected and cultured in DMEM (Gibco, Waltham, USA) supplemented with 10% fetal bovine serum (FBS) (Gibco, Waltham, USA), 100units/ml penicillin and 100μg/ml streptomycin. Up to approximately 80% confluences, cells were treated with 10 μM AZ3451 in the presence or absence of 10 ng/mL IL-1β. Passages 1–3 chondrocytes were used in our experiment to avoid the phenotype loss.

### Annexin V-FITC/PI staining

An Annexin V-FITC Apoptosis Detection Kit (Beyotime, China) was applied to measure the apoptosis rates based on the manufacturer’s instructions. In brief, collected chondrocytes were suspended in binding buffer with 1 × 106 cells/ml, and then incubate with 5μl Annexin V-FITC and 10μl PI in the dark for 15 min. The samples were analyzed using a FACScan flow cytometer (Becton Dickinson, USA).

### Measurement of mitochondrial membrane potential

A mitochondrial membrane potential assay kit with JC-1 (Beyotime, China) was applied to detect changes in mitochondrial membrane potential based on the manufacturer’s instructions. When the mitochondrial membrane potential is high, JC-1 can be collected in its matrix, and the aggregated JC-1 exhibits red fluorescence. When the mitochondrial membrane potential is low, JC-1 exists as a monomer, and the scattered JC-1 exhibits green fluorescence. All images were obtained using a fluorescence microscope.

### Western blot

For isolation of total cell extracts, cultured chondrocytes were harvested with RIPA lysis buffer (Boster, Wuhan) containing protease and phosphatase inhibitor cocktails. The extraction and isolation of nuclear and cytoplasmic protein were performed according to the Nuclear and Cytoplasmic Protein Extraction Kit (Beyotime, China). The protein concentration was determined using the bicinchoninic acid (BCA) protein assay (Boster, Wuhan, China). 25 μg protein samples were loaded on 8-12% SDS-PAGE and then transferred to polyvinylidene difluoride (PVDF) membranes. After blocking in 5% BSA for 1 h at room temperature, the membranes were then incubated with aforementioned primary antibodies overnight at 4°C. After incubation with the respective secondary antibodies for 1 h, the enhanced chemiluminescence (ECL) reagent (Boster, Wuhan, China) was applied to the bands and the intensity of the protein bands was quantified with Image Lab 3.0 software (Bio-Rad).

### Immunofluorescence

The translocation of P65 in nuclear and the expression of PAR2 in articular cartilage were quantified by immunofluorescence staining. 5-μm sagittal sections and chondrocytes were firstly fixed in 4% paraformaldehyde and penetrated in 0.1% Triton X-100, and then incubated with primary antibodies against P65(1:200) and PAR2(1:100) at 4 °C overnight. After rinsing with PBS for three times, the cells and sections were next incubated with anti-rat TRITC with a dilution rate of 1:100 (Boster, Wuhan, China) for 1h the darkness at room temperature. Finally, the nuclei were stained with DAPI for 5 min. All images were obtained using a fluorescence microscope. The rate of PAR2 positive cells each section was quantitated from 6 rats of each group.

### Transfection of tandem GFP-RFP-LC3 adenovirus

In order to detect autophagy flux, chondrocytes were transfected with mRFP-GFP-LC3 double-labeled adenovirus vectors (HanBio Technology, Shanghai, China). Autophagosomes (shown in yellow) and autolysosomes (shown in red) were performed with a Nikon C2+ laser scanning confocal microscope (Nikon America Inc., Melville, NY).

### SA-β-gal staining

SA-β-gal staining kit (Beyotime, Shanghai, China) was applied to measure the level of senescence following the manufacturer’s protocol. Senescent chondrocytes expressing SA-β-gal were stained blue.

### TUNEL staining

TUNEL assay was conducted to detect apoptotic cells using the one-step TUNEL apoptosis assay kit (Beyotime, Shanghai, China) based on the manufacturer’s instructions. The cells with red fluorescence were defined as apoptotic cells. The percentage of TUNEL-positive cells relative to DAPI-stained cells was calculated.

### Animal model

Eighteen male 8-week-old Sprague–Dawley rats were purchased from the Experimental Animal Centre of Tongji Medical College, China. The rat post-traumatic osteoarthritis (PTOA) model was induced by surgical destabilization of the anterior cruciate ligament (ACLT) on the right knee. The rats were randomly divided into three groups. The sham group (n=6) underwent sham operations with no ligament transection and treated with vehicle (physiological saline). The PTOA group (n=6) underwent operations and treated with vehicle. The PTOA/AZ3451 group (n=6) underwent operations and treated with AZ3451. For intra-articular injection, AZ3451 was dissolved in physiological saline (50μg/ml) and rats were given an intra-articular injection (100 μl) of AZ3451 or vehicle twice a week following surgery. Rats were sacrificed at 8 weeks post-OA surgery from each group.

### Histological assessment

The whole knee joints of rats were collected and fixed in 4% paraformaldehyde for 24 h. After decalcification in 10% EDTA solution for 30 days, the samples were embedded in paraffin, cut into 5 μm–thick sections, and stained with H&E, Toluidine blue and Safranin O/Fast Green. The sections were next subjected to immunohistological staining using antibodies recognizing MMP13 (Abcam, 1;200), Cleaved-caspase3 (Cell Signaling Technology, 1:200) and Beclin1(Proteintech Group, 1:200). The Osteoarthritis Research Society International (OARSI) grade system were used to evaluate histopathologic changes in osteoarthritic cartilage [44]. All scorings were performed by three independent observers in a blinded manner. The rate of positive cells each section was quantitated from 6 rats of each group.

### Statistical analysis

Data are presented as mean ± standard deviation (SD) from at least three independent experiments. All data were tested for normal distribution by Shapiro-Wilk test and for homogeneity of variances by Levene’s test. Data with normal distributions were compared using one-way analysis of variance (ANOVA) followed by Tukey’s post-hoc test. Nonparametric data (OARSI scores) were analyzed by the Kruskal–Wallis H test. Results were considered significant for P-values< 0.05.
